# Ophthalmic Surgery

**Published:** 1888-06

**Authors:** 


*Ophthalmic Surgery.* By R. BRUDENELL CARTER, F.R.C.S., and W. A. FROST, F.R.C.S. London: CASSELL & CO. 1887.

The book before us well maintains the excellent reputation of *The Clinical Manuals for Practitioners and Students of Medicine.* Our authors are well-known ophthalmic surgeons, thoroughly competent to write upon their speciality, both from large practical experience and from wide literary knowledge of it.

This volume is very readable. Its contents are arranged in fifteen chapters, for each one of which one of the authors is responsible; but the whole work has been submitted to both.

Full use has been made of the space at their command; each chapter is complete in itself, and little of importance appears to be omitted: a copious index gives easy reference to particular points.

The scope of medicine is so wide, that the student does well if in his academical career he gain a really sound grasp of its general principles, together with a good knowledge of anatomy and physiology. But the practitioner has to deal with disease in all its varied forms, and patients with eye trouble will assuredly come for advice. The eye is an organ of no less importance than many others, and some knowledge of the diseases to which it is specially subject is very soon found to be essential.

Unhappily it too often occurs that where no evident lesion presents itself, and even where definite inflammation exists, eye cases are diagnosed as "constitutional," and are treated on general principles, no special attention being given to the organ affected: prescriptions are written for the neuralgia or rheumatism, while the eye is being spoiled by insidious iritis or glaucoma.

Our authors have produced a book which any senior student could read and understand; it is very well suited to the needs of the practitioner, and may be recommended as a sound introduction to the study of ophthalmic surgery.

